# Chemical Safety and Quality Attributes of Dried Sausage Snacks Stored in Modified Atmosphere

**DOI:** 10.1155/2022/6173613

**Published:** 2022-12-14

**Authors:** Jagoda Majcherczyk, Ireneusz Maciejaszek, Krzysztof Surówka

**Affiliations:** Department of Biotechnology and General Technology of Food, Faculty of Food Technology, University of Agriculture in Krakow, 122 Balicka Street, 30-149 Cracow, Poland

## Abstract

A sausage snack was produced by air blast drying of cottage sausage, and its safety and quality were investigated during 10-week storage period in a modified atmosphere (20% CO_2_/80% N_2_) at 4 ± 1°C and 20 ± 1°C. The results of a sensory and instrumental evaluation of colour and texture showed that significantly greater changes in these parameters were caused by drying the sausage rather than during subsequent storage of the produced snacks. Although total viable count increased and reached a maximum value of 6.5 log CFU g^−1^ after 70 days of storage at 20 ± 1°C, *Enterobacteriaceae* were not detected. The histamine level was approx. 18 mg kg^−1^, while the average content of the other biogenic amines after 70 days of storage ranged from 4.4 mg kg^−1^ (cadaverine) to 32.1 mg kg^−1^ (tyramine). The biogenic amine index was small and varied from 34.5 mg kg^−1^ (in the sausage before drying) to 73.4 mg kg^−1^ (at the end of the snack storage at 20 ± 1°C). The lipid hydrolysis and oxidation proceeded slowly and did not pose a threat to the product quality. The combination of drying and modified atmosphere packaging maintains good quality and safety of sausage snack during 70 days at refrigerated storage and/or 4 weeks at room temperature.

## 1. Introduction

Snack food products have found a solid place among consumers, and along with contemporary busy lifestyles, they constitute an important, steadily increasing segment of food product market. Their consumers are people of almost all ages, but the current growth in their popularity is associated primarily with the generation of “millennials,” looking for an alternative to traditional meals that are suitable for satisfying hunger during the day [1, 2]. However, the vast majority of these are products of plant origin, such as potato chips or corn meal extrudates, while snacks made from animal raw materials are much less popular. These include beef jerky originated from the USA; dried seafood from the Far East, such as Japanese dried squid rings (Ika Kun); and dried fish, eagerly eaten in Eastern Europe as snacks [3–5]. We think that completely new snack products could also be made from sausage.

Many sausages are traditional and highly appreciated products manufactured in numerous countries worldwide [6, 7]. Most often, they are the main ingredients of meals. However, they can be processed into ready-to-eat (RTE), high quality, and nutritious dried meat products—sausage snacks. Apart from the main benefits such as easy accessibility and characteristic sensory qualities, producers of RTE meat products must follow stringent food safety guidelines to prevent the risk of diseases [8]. It is important to evaluate the impact of product preparation, packaging, and different conditions of storage on the safety and quality parameters of final products with special reference to chemical indices such as accumulation of biogenic amines (BAs) and lipid oxidation, microbiological quality, and sensory properties [9]. It is believed that the level of biogenic amines could serve as a good chemical indicator of the quality of sausage snacks [10].

Biogenic amines are basic nitrogenous compounds formed by amino acid decarboxylation or by amination and transamination of aldehydes and ketones. They can be found in fresh meat at relatively low concentrations. In fresh pork meat, only a higher level of spermidine and spermine was observed [11]. However, storing meat even in refrigerated conditions causes an increase of the amine concentration [12] caused by the contaminating microflora exhibiting amino acid decarboxylase activity [13–15]. Research on biogenic amines is of particular interest in the case of sausages. In traditional ripening sausages from various European countries, the main amines are tyramine, putrescine, and cadaverine, although large differences were found in terms of the country of origin, technology used, and the raw material composition [7, 16]. Furthermore, during storage, the concentrations of free amino acids, precursors of BAs, increase as a result of the proteolytic event, primarily due to the presence of many microorganisms [11]. The consumption of food containing large amounts of BAs can have toxicological consequences for consumers, including an increase of blood pressure, migraines, and even haemorrhages [17]. At present, there are no regulatory standards for BA toxic levels. Histamine and tyramine are considered as the most toxic. The Food and Drug Administration (FDA) recommends lower histamine levels in fish and fish products, up to 50 mg kg^−1^ for healthy individuals, whereas the level of 600 mg of tyramine per meal has no adverse effect on a nonsusceptible person [18]. The most toxic consequence of BA intake is histamine poisoning, whose symptoms, apart from these aforementioned, are the digestive system disorders, headaches, and rash [19].

An effective method of extending the shelf life, including the inhibition of BA formation, is the use of refrigeration and proper packaging. Modified atmosphere packaging (MAP) and vacuum packaging contribute to extending the shelf life by inhibiting moisture loss, retarding lipid oxidation, and preventing microbial growth. The use of MAP packaging technology is becoming more and more extensive, as it allows gas composition to be optimised in order to ensure both product quality and safety. In the commonly applied atmospheres, the most important component is carbon dioxide (CO_2_) due to its antimicrobial activity, whereas N_2_ is a filler [20, 21]. The benefits of this method in the storage of dry-cured meat products have been confirmed by numerous authors [22–24]. Ameer et al. reported that MA packaging of dry-fermented sausages was found to be more effective than vacuum package. Several examined quality parameters confirmed that extending the shelf life had no effect on sensory quality characteristic during storage [25].

Previous studies have shown that traditional Polish cottage sausage, one of the most recognizable traditional products in Central Europe, is characterized by the properties that make it suitable for the production of sausage snacks [26]. This sausage is a smoked, mildly cured, medium-minced meat product made of pork meat and fat, occasionally with added beef, highly seasoned with pepper and garlic, and stuffed into natural small intestine casings. Therefore, the aim of this study was to determine the impact of air blast drying on cottage sausage properties and evaluation of dried sausage snacks produced in this way in terms of their safety and qualitative changes during MAP storage under various temperature conditions. It was hypothesised that the obtained product should be characterized by good storage stability due to the effect of hurdle technology combining such factors as smoking, curing, presence of allicin, terpenes, and flavones in garlic and pepper used as spices, drying, MAP packaging, and cooling.

## 2. Material and Methods

### 2.1. Dry Sausage Snack Processing and Sampling Procedures

Cottage sausage was produced in a medium-sized meat processing plant in the Cracow (Poland) area according to the traditional recipe and technology previously described [26]. On the next day, it was cut into slices 6 mm thick with the skin left on and air blast dried for 24 hours at 40 ± 1°C, with the speed of approx. 1 m s^−1^, using a Stöckli Dehydrator, model 0076.72 M, Switzerland, with stainless steel trays, 33.5 cm in diameter, and thermostatic control from 20°C to 70°C. The sausage slices were manually turned every 8 h during drying. The dried sausage snacks, produced in this way, were cooled, and 52 ± 2 g portions were packed in polyethylene/polyamide bags (PE/PA 50/20) with dimensions of 19.0 × 25.0 cm (El-Pack Ltd., Cracow, Poland), which according to the test methods, DIN 53380 and DIN 53122 showed a permeability to CO_2_ and O_2_ of 150 and 50 mL/m^2^/24 h, respectively, N_2_ permeability of 10 mL/m^2^/24h, and water vapour transmission of 3 g/m^2^/24 h at 23°C according to the manufacturer. A Boxer 42 chamber packaging machine (Henkelman, Hertogenbosch, Netherlands) and 20% CO_2_/80% N_2_ gas mixture (pressure 175 mbar) (Linde Gaz Polska, Cracow, Poland) were used. The ratio of headspace to product was approx. 4 : 1. Packages were stored in the dark at 4 ± 1°C and 20 ± 1°C in CLN 32 SMART and CHL 1 B SMART laboratory incubators, respectively (POL-EKO-APARATURA, Poland). The analyses were carried out for both freshly prepared sausage and dried sausage snacks. Fourteen packs were used for each temperature treatment (2 pieces for each analysis day, 7 storage periods). The packs with products were taken for subsequent analysis after 0 (sausage snack just after drying), 3, 7, 14, 28, 56, and 70 days of storage.

### 2.2. Proximate Composition and Physicochemical Measurements

Water content was determined by drying at 103°C to a constant weight using an oven SLN 32 ECO (POL-EKO-APARATURA, Poland) [27]; ash at a combustion temperature of 550°C in a laboratory chamber furnace L 05/12 (LAC, Czech Republic) [28]; protein by the Kjeldahl method [29]; and total lipids using the Soxhlet method [30]. The pH measurement was conducted using an HI 9321 pH meter (Hanna Instruments Woonsocket RI, USA) according to ISO Standard [31] by inserting the combined pH electrode (Elmetron, Poland) directly into the sample : water (1 : 10 *w*/*v*) homogenate, whereas water activity (*a*_*w*_) was analysed at 25°C using a LabMaster-aw meter (Novasina, Zurich, Switzerland). For each parameter, at each time and storage temperature, the results were calculated as the mean value of three replications.

### 2.3. Sensory Analysis

Sensory analysis was performed by a 9-member panel of assessors, appropriately trained according to the Polish Standard and thoroughly familiar with the definition and intensity of each of the quality descriptors [32]. The following attributes were assessed: surface and cross-section appearance, texture, and odour and taste using a 5-point scale, where 5 is the best and 1 is the worst. The score 5 (the best) was assigned for individual attributes as follows: surface and cross-section: smooth and evenly coloured; texture: firm and chewy; and odour and taste: intensive and strongly desired. The attributes of the worst product (score 1) were strongly folded and poorly coloured surface, particles in cross-section frayed, and extremely unevenly disposed; definitely too tough and extremely dry texture; and undesirable (strange) odour and taste. The acceptability threshold was established at 3.0 points. The criteria for this score are surface: creased with irregular colour; cross-section: irregularly coloured and unevenly disposed particles; texture: not even, too tough, or too loose; and odour and taste: perceptible, less intensive, and neutral. The results obtained by 9 assessors for particular quality attributes were summed, and the means ± SD were calculated. The overall sensory quality (OSQ) was expressed as a mean of the scores gained by all particular attributes; however, when at least one attribute was rated below 3.0, the OSQ < 3.0.

### 2.4. Instrumental Colour and Texture Analysis

Colour was objectively measured on sliced samples, situated on the Minolta CM-5 Chroma meter (Minolta, Ramsey, NJ, USA) plate with an 8 mm diameter measuring hole with specular component excluded (SCE) mode. The following colour parameters in the CIE,*L*^∗^*a*^∗^*b*^∗^space under the illuminant D65/10°, were recorded:*L*^∗^ (*L*^∗^ 100 = white and *L*^∗^0 = black), *a*^∗^ (redness (+) and greenness (-)), and *b*^∗^ (yellowness (+) and blueness (-)). Total colour differences (Δ*E*) were calculated using

(1) $$\begin{array}{c} \Delta E=\sqrt{{\left(\Delta L^{*}\right)}^{2}+{\left(\Delta a^{*}\right)}^{2}+{\left(\Delta b^{*}\right)}^{2},} \end{array}$$

where *L*^∗^, *a*^∗^, and *b*^∗^ values of the product just after drying (storage time 0 days) were chosen as the basis reference for the colour comparison [33].

Texture profile analysis (TPA) was performed using a TA-XT2 Texture Analyser (Stable Micro Systems Ltd., Godalming, Surrey, UK) on 20 mm diameter samples cut from fresh sausage as well as dried and stored snacks. The thickness of the fresh sausage was 6 mm, whereas thickness of dried and stored snacks was smaller as it decreased during drying. The double compression test with an SMS P/45 plunger was carried out at a rate of 0.1 mm·s^−1^ and the time between strokes 10 s to compress the sample to 50% of its original height. Changes in force over time were recorded and used to determine the hardness, springiness, cohesiveness, and chewiness of the tested material [34]. For fresh sausage and stored sausage snacks, at each time and temperature, colour was analysed in triplicate and texture in six replications and the mean values were calculated.

### 2.5. Fat Rancidity Determination

Acid value (AV) (mg KOH g^−1^ of extracted fat) was determined based on the Polish and International Standards [35, 36]. Briefly, 40 g of finely crushed sample was homogenized with 200 mL of a 3 : 1 chloroform : methanol mixture, shaken vigorously in a separatory funnel for 10 minutes and left to allow the phases to separate. The chloroform extract (bottom layer) was filtered through Whatman No. 1 filter paper. From the filtrate, 50 mL was taken and titrated with 0.1 M KOH for visual titration. Simultaneously, the 50 mL of filtrate was put into a weighing glass to determine the fat content of the extract by weight.

Peroxide value (PV) (meq O_2_·g^−1^ of extracted fat) was determined according to the International Standard with modifications [37]. Fat extract was obtained from the finely crushed sample (20 g) by homogenization with 100 mL of chloroform (MICROTRON MB 550, Kinematica AG, Switzerland). The mixture was filtered through Whatman No. 1 filter paper, and 20 mL of filtrate was put into a stoppered conical flask and mixed with 30 mL of glacial acetic acid and 1 mL of saturated potassium iodide. The released iodine was titrated with 0.01 M sodium thiosulfate. Fat content of the extract was determined by the gravimetric method.

Thiobarbituric acid reactive substances (TBARS) (mg malondialdehyde (MDA)·kg^−1^ of product) were determined as described by Ahn et al. [38] and 1,1,3,3-tetraethoxypropane (Sigma-Aldrich GmbH) was used as a standard. For each parameter, at each time and storage temperature, the results were calculated as the mean value of three replications.

### 2.6. Microbiological Analysis

All microbiological analyses were performed according to the International Commission on Microbiological Specifications for Foods (ICMSF, 2011) [39]. The sample (10 ± 0.1 g) was mixed with 90 mL of Ringer's solution (2.25 g·L^−1^ NaCl, 0.105 g·L^−1^ KCl, 0.12 g·L^−1^ CaCl_2_·6H_2_O, and 0.05 g·L^−1^ NaHCO_3_), and the whole was homogenized for 2 min in a Stomacher (IUL Instruments, Barcelona, Spain). Further decimal solutions were prepared from the same dilution. The total viable count (TVC) was determined on plate count agar (Merck, Darmstadt, Germany) after incubation at 30°C for 72 h. Lactic acid bacteria (LAB) were assayed using de Man-Rogosa-Sharpe (MRS) agar (Oxoid, Hampshire, UK) in compliance with the instructions provided by the producer. Determination of the *Enterobacteriaceae* count was performed on crystal violet neutral red bile glucose agar (VRBG), incubated at 37°C for 24 h. The detection limit of the above techniques was 10 colony-forming unit per gram (CFU/g) except for LAB and *Enterobacteriaceae*, whose limit was 10^2^ CFU/g.

### 2.7. Determination of Biogenic Amine Content

Concentrations of eight biogenic amines (2-phenylethylamine, cadaverine, histamine, putrescine, spermidine, spermine, tryptamine, and tyramine) were measured by trichloroacetic acid (TCA) extraction, derivatization with benzoyl chloride (Sigma-Aldrich Co., Darmstadt, Germany), and HPLC quantification according to Özogul et al. [40] with slight modifications. A 5 g sample was homogenized (Heidolph, Schwabach, Germany) with 20 mL of 6% TCA and then centrifuged at 10 000 × *g* for 10 min in a MPW-54 centrifuge (MPW Med. Instruments, Warsaw, Poland) and filtered. The collected filtrate was diluted to 50 mL with distilled water. Subsequently, a 2 mL aliquot of sample was spiked with 1 mg mL^−1^ serotonin (50 *μ*L) as an internal standard and alkalinised with 1 mL of 1 M NaOH (Chempur, Piekary Śląskie, Poland). For derivatization, 20 *μ*L of benzoyl chloride was used, and the reaction mixture was kept at 24°C ± 1°C for 30 min. The process was stopped by adding 2 mL of saturated NaCl (Chempur), and the mixture was extracted twice with 2 mL of diethyl ether. Then, combined organic layers were evaporated to dryness in a nitrogen stream. The residue was dissolved in 1 mL of acetonitrile (Merck, Germany), filtered through 0.45 *μ*m syringe filter (Merck Millipore, Darmstadt, Germany), and 20 *μ*L was injected on the column. HPLC analysis was performed using the Merck Hitachi LaChrom HPLC System (Tokyo, Japan) with a diode array detector. Separation of analytes was carried out on an ACE 3 C18 column (150 × 4.6 mm) at 30°C by using gradient elution with acetonitrile (ACN) and HPLC grade water (Polwater, Poland). The elution started with 40% of ACN, ramped at 75% (13 min) and 100% (3 min) with a flow rate from 1 mL min^−1^ to 1.5 mL min^−1^ in 16 min. Detection was conducted at 254 nm. Calibration curves for each of the amine standards were prepared in the range 2-100 *μ*g mL^−1^. Quantitative analysis of biogenic amines was performed by an external calibration curve method with serotonin as the internal standard (50 *μ*g mL^−1^). The biogenic amine index (BAI) was calculated as the sum of histamine, putrescine, cadaverine, and tyramine concentrations [41]. For each parameter, at each time and storage temperature, the results were calculated as the mean value of three replications.

### 2.8. Statistical Methods

The results obtained were analysed statistically using CSS Statistica v. 13 software (StatSoft Polska, Krakow, Poland). The significance of differences was determined by 2-way analysis of variance (ANOVA) with Student-Newman-Keuls' multiple range test. The factors analysed were temperature of packaging with two levels and days of storage with seven levels. A value of *p* < 0.05 was used to indicate significant differences.

## 3. Results and Discussion

### 3.1. Changes in Chemical Composition, pH, and Water Activity

Freshly prepared sausage contained 50.4 ± 0.59% moisture, 21.2 ± 0.37% protein, 25.8 ± 0.88% fat, and 2.6 ± 0.1% ash. After 24 hours of drying, the slices of sausages lost weight mainly due to water evaporation and partially as a result of fat rendering. As a consequence, the amount of moisture decreased to 14.3 ± 0.42%, and lipid, protein, and ash contents increased to 35.1 ± 0.42%, 45.1 ± 0.21%, and 5.5 ± 0.14%, respectively.

Starter cultures were not used in the production of cottage sausage, and meat was from the next day after slaughtering. The pH of the sausage obtained was slightly acidic (pH 6.12 ± 0.007), characteristic for unfermented sausages [26]. As a result of drying, its value did not change significantly (*p* > 0.05). During the storage at 4°C, the final pH value was 6.14 ± 0.006, while at 20°C, it reached a maximum of 6.16 ± 0.010. Other authors [42, 43] reported a similar trend for drying meat products, in which there were no significant differences in pH values during storage periods. According to Bover-Cid et al. [44], in some ripened meat products processed at low temperatures, fermentation is limited, and thus, the pH does not decrease by more than 0.2-0.4 units. Invariability of pH during storage might be caused by the balancing of two different phenomena: decrease of pH related to organic acid production by LAB and its increase due to the liberation of metabolites resulting from bacterial activity [45, 46].

Water activity (*a*_*w*_) is an important safety factor during the storage of meat products. Low values can limit the growth of microorganisms ensuring the safety of a product, whereas extremely low water activity can lead to a hard texture and poor flavour [47]. The initial water activity of fresh sausage was 0.963 ± 0.001, but after drying, it decreased substantially to 0.792 ± 0.011. During the subsequent storage, the changes of water activity at both temperatures were almost unchanging and statistically insignificant (*p* > 0.05). This agree with the findings of Rubio et al. [46], who studied the influence of storage period and packaging effect on sliced dry-cured beef. The authors observed that water activity of the samples preserved in a gas mixture (20% CO_2_/80% N_2_) did not change during storage for 210 days at 6°C. Also in dried beef and pork packed loosely and stored for 30 days, the water activity did not change statistically significantly and ranged from 0.835 to 0.794 [48].

### 3.2. Sensory Analysis

Table 1 illustrates changes in individual sensory attributes and overall sensory quality (OSQ) of dried sausage snacks over the 70-day storage in MAP (20% CO_2_/80% N_2_). As all sensory attributes of freshly prepared sausage were scored highest (5.0) and the OSQ of the product immediately after drying was 4.9, it can be seen that the drying process did not negatively affect the quality. The applied temperature of 4°C combined with MAP well protected the product against any serious sensory changes for the whole storage period. At the end of storage, all sensory features were scored highly. In turn, texture, taste, and odour contributed more to the decrease in OSQ than surface and cross-section appearance. During storage at 20°C, sensory changes were running faster than at 4°C; after a week, a statistically significant difference was found for taste, after two weeks for the odour and cross-section appearance and after four weeks for surface appearance as well. Since the taste and odour rating was below the threshold (3.0), after 56 days of storage, the tests performed showed that the dried sausage snacks packed in MAP at 20°C maintained sensory acceptability for 28 days. Thus, it can be concluded that the modified atmosphere in which the samples were stored only partially protects the product against adverse changes in sensory characteristics. Therefore, in order to ensure a long storage period, a temperature reduction should be applied at the same time. Such an approach in the case of processed meat is fully rational and applies also to low-moisture products such as dry-fermented sausages or dry-cured hams [49]. Similarly, the recommended temperature for storage of pastirma, one of the oldest dry-cured intermediate moisture meat products of Middle Eastern countries, is under 10°C [50].

### 3.3. Instrumental Colour Analysis

Consumers often choose a specific product based on its appearance, which is usually largely determined by colour.  Table 2 shows the CIE-LAB colour parameters of the freshly prepared sausage and dried sausage snack during its storage. As a result of drying, a strong decrease of lightness (*L*^∗^ value) was observed. The opposite tendency can be seen during the storage of the dried product at both temperatures. At 4°C, significant differences between measurements were observed on the 56^th^ day, whereas for samples stored at 20°C, the first significant changes were observed on the 14^th^ day. The trend towards growing *L*^∗^ values during storage is similar to those detected by Piras et al. [51] in dry-cured ham slices. Redness (*a*^∗^ value) is used as an indicator of colour stability in meat products [24]. As a result of the drying process, this parameter slightly but significantly increased, which is probably due to the partial loss of water and fat. During the storage period, no major changes were detected, but the final *a*^∗^ value at 4°C was higher, while at 20°C, it is lower than at the beginning. On the other hand, yellowness (*b*^∗^ value) decreased after drying, while during storage at both temperatures, it remained at the same level until the last two measurements, where a growing tendency was observed. The decrease in the *L*^∗^ value due to drying and changes in the *a*^∗^ and *b*^∗^ parameters are associated with the removal of some water and fat from the product, which changed the characteristics of light reflection from it. Further colour changes occurring during the storage of sausage snacks could be caused by the equalization of moisture in the entire mass of the packed product after drying, crystallization of the fat fraction, and changes taking place within the muscle pigments.

The measure of change in visual perception (Δ*E*) between the freshly obtained sausage snack (storage time 0 days) and the sausage from which it was made equals almost 20 and is in the range of 11-49, which according to a commonly accepted criterion [52] indicates that these products differ significantly in colours but still have common features. On the other hand, the fact that up to 7 days Δ*E* < 1.0 proves that during this time no colour changes occur that are perceptible to the human eye, but after that period, they are perceptible through close observation (1 < Δ*E* ≤ 2) and even at a glance (2 < Δ*E* ≤ 10).

### 3.4. Textural Analysis

In this study, it was assumed that the drying process would change the textural parameters of sausage so that the obtained product would have increased chewiness demanded by the consumers from snacks. Actually, in addition to the loss of water, some fat is also melted during the drying process, which significantly contributes to changes in the texture. This, in turn, results in about fourfold increase in average hardness and chewiness (Table 3). The subsequent storage of the sausage snack led to some changes in these parameters. They were, however, smaller, although the hardness and chewiness of the product stored at 4°C increased compared to the initial values by 11 and 14% and at 20°C by 15 and 20%, respectively. A similar increase in hardness during the 60-day storage of beef jerky was reported by Jung et al. [53]. Springiness and cohesiveness also changed as a result of the drying; springiness decreased by 13%, while cohesiveness increased by 7%. During storage, both these parameters fluctuated slightly, and their values up to 56^th^ day of storage did not differ significantly from those recorded for the product just after the drying process. The instrumental texture profile analysis performed here confirms the results of sensory evaluation and leads to the conclusion that the texture changes are not the cause of the deterioration of the quality of the sausage snack during its storage at both temperatures used.

### 3.5. Fat Rancidity Determination

In medium-fat sausages, including Polish traditional cottage sausages, lipid oxidation may be a factor limiting their shelf life. The level of the product quality decline due to lipid hydrolysis and oxidation throughout drying and storage was evaluated by determining acid value (AV), peroxide value (PV), and the 2-thiobarbituric acid-reactive substance (TBARS) index (Table 4). Throughout the drying process of the sausage, AV decreased by about 14%. It could have resulted from a change in the composition of the lipid fraction caused by the loss of approx. 12% of fat as a result of its rendering during drying. At the same time, PV increased by about 69%, and there were no significant changes in the level of TBARS. AV hardly changed up to the 14^th^ day of storage, but it grew more dynamically after that. A similar upward trend was also observed for PV, which was initially slower and then from the 28^th^ day clearly faster, especially at 20°C. This tendency also applies to the TBARS value, except that it grew steadily throughout the whole period of storage of the sausage snack. The results presented here show that among the analysed fat quality indices, AV slightly exceeded 4 mg KOH·g^−1^ at the end of the storage at 20°C. The Codex Alimentarius states that the AV of fats and oils should not be greater than this level and the maximum allowable PV value is 10.0 meq O_2_·kg^−1^ [54]. The TBARS value does not have standardized criteria, but it is widely accepted that the level up to 5 mg MDA kg^−1^ indicates good product quality [55]. Undoubtedly, the oxygen-free packaging of the sausage snack in MAP significantly contributed to slowing down the unfavourable changes in fat. In addition, the smoking process that was used in the sausage production, and inclusion in the recipe of garlic and pepper, which contain natural antioxidants such as allicin, diallyl sulfides, and other sulfur compounds, also played an important role [56–60].

### 3.6. Microbiological Quality

**Figure 1:**
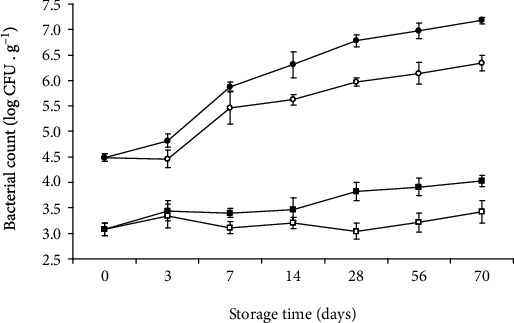
Total viable count (TVC) and lactic acid bacteria (LAB) in sausage snack stored in MAP (20% CO_2_/80% N_2_) at 4°C and 20°C. Description of symbols: white circles indicate TVC at 4°C; black circles indicate TVC at 20°C; white squares indicate LAB at 4°C; black squares indicate LAB at 20°C. Error bars indicate standard deviation.

Microorganisms present in meat products have a crucial impact on their safety; moreover, their presence also plays a particularly important role in shaping quality and sensory characteristics [61]. The initial total viable count (TVC) and lactic bacteria (LAB) contamination of the fresh sausages were 3.63 log CFU g^−1^ and 2.97 log CFU g^−1^, and after the drying process, both values increased to 4.49 log CFU g^−1^ and 3.08 log CFU g^−1^, respectively. This is probably caused by loss of humidity, concentration of dry matter, and the microflora-friendly temperature (40°C) of the air used as the drying medium. During the first week of storage TVC, mean counts were similar at both temperatures. A significant increase was detected on the 14^th^ day (Figure 1) in samples stored at 20°C, and the level of 6.32 log CFU g^−1^ was reached at the end of the storage, whereas for samples stored at a lower temperature, the slow and systematic growth of TVC was observed to approximately 5.97 log CFU g^−1^. *Lactobacillus* species occur during fermentation, ripening, and drying processes [62]. In the present study, the count of LAB in dried sausage snacks stored under MAP showed no significant changes at both temperatures. The constant level of LAB was due to water activity below 0.8, which is the limiting value for the growth of these microorganisms [63]. *Enterobacteriaceae*, considered to be a hygienic indicator, were under the detection limit (10^2^ CFU/g) in all samples at both temperatures, which may be due to low water activity and was confirmed by other authors, who analysed MAP packaged dry-cured ham [24].

### 3.7. Biogenic Amine Contents

Six biogenic amines were detected in the sausages before drying, namely, histamine, putrescine, spermidine, spermine, tryptamine, and tyramine (Table 5). All of them increased in concentration as a result of drying, and consequently, the biogenic amine index (BAI) also increased by 68%. This could be due to the action of decarboxylases. It seems, however, that the main reason for this phenomenon is the removal of part of the water and the resulting increase in the concentration of nonaqueous components, including BAs. Despite the different storage temperatures, the concentrations of individual biogenic amines did not differ statistically significantly for most of the storage time. Such differences were only found for tyramine on the 28^th^ day, tryptamine on the 56^th^ day, and cadaverine, histamine, putrescine, and spermidine after 70 days. It follows that in the case of sausage snacks—a low-moisture product—refrigeration no longer reduces the formation of amines, since the growth of microorganisms, which might be involved in amine formation, is anyway limited by the low water activity, which is almost 0.8. This value is in the range of 0.6 to 0.9, typical for shelf-stable products classified as intermediate moisture food (IMF) [64].

Changes in amine concentrations over the 10-week storage period were generally small and often statistically insignificant. The histamine, commonly considered to be the most dangerous for consumers, was detected at a level of approx. 18 mg·kg^−1^. If we assume that a single consumed portion of the snack product described here weighs about 100 g, the histamine intake in a single meal would be far less than 50 mg/person/meal—a quantity which is recognized by the European Food Safety Authority (EFSA) [18] as not having a harmful effect on healthy people. However, for people with histamine intolerance, even a small amount can cause diarrhoea, headache, and other symptoms [65]. Although enzymes and microorganisms are active during maturation, which occurs during the manufacture of many sausage varieties, there is generally no significant accumulation of histamine. This was confirmed by Latorre-Moratalla et al. [7], who noted that histamine content was greater than 10 mg·kg^−1^ only in 20% of different European sausages studied in the Tradisausage project. In only two cases, the level of histamine exceeded 100 mg·kg^−1^. In addition, Surówka et al. [26], who analysed cottage sausage produced in 24 randomly selected meat processing plants in Poland, also reported that although histamine was found in 42% of sausages, its level amounted to 2.6-34.2 mg·kg^−1^ and hence did not pose a threat to human health. A much higher risk of histamine intake may occur after consumption of certain fishery products [66] and maturing cheeses [67].

Putrescine and cadaverine are biogenic amines which are able to potentiate the toxic effect of histamine by inhibiting intestinal histamine-metabolizing enzymes. In addition, they can react with nitrite to form cancerogenic nitrosamines [68]. Fortunately, there was no cadaverine in the product until the third day of storage; later, it was detected only at levels of about 4.5 mg·kg^−1^. Although the concentration of putrescine was more than three times higher, it was still distinctly lower than in European fermented sausages, which have a median value of putrescine of 51 mg·kg^−1^.

Tyramine is the most abundant amine in ripened meat products, but its content varies greatly [8]. Of all amines, its concentration during storage tests of sausage snack was the highest, approx. 32 mg·kg^−1^. According to Brink et al. [69], the tyramine contents from 100 to 800 mg·kg^−1^ should be respected for health and safety reasons. In turn, according to the EFSA [18], a tyramine content of 600 mg·kg^−1^ is safe for healthy consumers. However, for people taking 3^rd^ generation MAOI drugs, amounts as low as 50 mg·kg^−1^ can be harmful. As can be seen, the tyramine concentration in the tested snacks is so low that it is unlikely to pose a toxicological risk even for people particularly susceptible to its effects. It is worth noting, however, that there are sausages in which the concentration of this amine is as high as approx. 700 mg·kg^−1^ [70]. The content of tryptamine and 2-phenylethylamine, whose precursors are, respectively, tryptophan and phenylalanine, can also be considered as safe. The former was detected in low concentrations in starting sausage and in slightly higher concentrations in the sausage snack, while the latter was found only at the very end of storage at 20°C. As Suzzi and Gardini report [16], 2-phenylethylamine occurs sporadically in sausages, and if at all, its level is low. This is beneficial, as this amine, like tyramine, is considered to be an indicator of hypertensive crisis and dietary-induced migraine.

Both spermine and spermidine are amines which are naturally present in fresh pork meat [11]. According to some authors, they are not toxicologically important and their formation is not related to bacterial spoilage [41]. In the present study, the concentration of spermine was about twice that of spermidine, and both concentrations fluctuated slightly during snack storage.

The biogenic amine index, which consists of concentrations of amines with the greatest potential impact on food safety, increased by 16% and 26% during the storage of the analysed snacks at 4°C and 20°C, respectively. The increase was the highest between the 3^rd^ and the 7^th^ day of storage and was mainly caused by an abrupt increase in the concentration of tyramine and cadaverine during this period. The value of this indicator, comparable with the values presented in the literature for various sausages [71] and other food products [72, 73], clearly shows that the biogenic amines present in dry sausage snacks should not pose a risk.

## 4. Conclusions

Sausage snacks with attractive sensory characteristics can be made from traditional cottage sausage by air blast drying. Reduction in *a*_*w*_, combined with the modified oxygen-free atmosphere packaging used in this study, allowed the products to maintain their high quality for at least 70 days when stored at 4°C. Product kept at room temperature (20°C) was stable for a period of 4 weeks, which probably resulted from the nature of the raw material, containing natural antioxidants in spices and having undergone curing and smoking. The combination of all these elements within the hurdle technology explains the shelf life of the obtained snack product. There was also no risk from *Enterobacteriaceae*. The level of biogenic amines, including histamine, was definitely safe, much lower than in fermented sausages produced in European countries.

## Figures and Tables

**Table 1:** Changes in sensory attributes of sausage snack stored in MAP (20% CO_2_/80% N_2_) at 4°C and 20°C.

Storage time (days)	Storage temperature											
	4°C						20°C					
	Surface	Cross-section	Texture	Odour	Taste	OSQ^∗^	Surface	Cross-section	Texture	Odour	Taste	OSQ
0	4.8 ± 0.3	4.7 ± 0.3	5.0 ± 0.0^a^	4.9 ± 0.2^a^	4.9 ± 0.2^a^	4.9 ± 0.2^a^	4.8 ± 0.3^a^	4.7 ± 0.3^a^	5.0 ± 0.0^a^	4.9 ± 0.2^a^	4.9 ± 0.2^a^	4.9 ± 0.2^a^
3	4.7 ± 0.4	4.8 ± 0.3	4.9 ± 0.2^a^	4.7 ± 0.3^ab^	4.9 ± 0.2^a^	4.8 ± 0.2^a^	4.7 ± 0.3^a^	4.7 ± 0.3^a^	4.7 ± 0.3^a^	4.6 ± 0.3^ab^	4.5 ± 0.3^b^	4.6 ± 0.2^ab^
7	4.7 ± 0.3	4.7 ± 0.2	4.7 ± 0.3^ab^	4.4 ± 0.2^bc^	4.6 ± 0.2^abA^	4.6 ± 0.2^a^	4.7 ± 0.3^a^	4.7 ± 0.3^a^	4.4 ± 0.2^b^	4.1 ± 0.2^b^	4.0 ± 0.3^bB^	4.4 ± 0.2^b^
14	4.6 ± 0.4	4.6 ± 0.3^A^	4.4 ± 0.2^b^	4.2 ± 0.3^cA^	4.5 ± 0.3^bA^	4.6 ± 0.3^abcA^	4.1 ± 0.3^b^	4.0 ± 0.1^bB^	4.2 ± 0.3^bc^	3.5 ± 0.3^cB^	3.8 ± 0.3^bcB^	3.9 ± 0.1^cB^
28	4.7 ± 0.3^A^	4.7 ± 0.4^A^	4.2 ± 0.3^bc^	4.1 ± 0.2^cA^	4.4 ± 0.2^bA^	4.5 ± 0.1^bcdA^	4.0 ± 0.3^bcB^	3.9 ± 0.3^bB^	4.1 ± 0.2^c^	3.1 ± 0.2^cB^	3.7 ± 0.3^cB^	3.8 ± 0.2^cB^
56	4.6 ± 0.4^A^	4.6 ± 0.3^A^	4.1 ± 0.2^c^	3.9 ± 0.2^cA^	4.1 ± 0.2^cA^	4.4 ± 0.2^cdeA^	3.7 ± 0.3^cB^	3.7 ± 0.4^bB^	3.9 ± 0.2^c^	2.7 ± 0.2^dB^	2.8 ± 0.2^dB^	< 3.0^dB^
70	4.5 ± 0.4^A^	4.7 ± 0.3^A^	3.9 ± 0.2^c^	3.9 ± 0.2^cA^	3.9 ± 0.2^cA^	4.3 ± 0.2^dA^	3.6 ± 0.3^cB^	3.7 ± 0.4^bB^	3.8 ± 0.3^c^	2.6 ± 0.3^dB^	2.7 ± 0.3^dB^	< 3.0^dB^

Mean values in columns marked with the same superscript or not marked at all do not differ significantly (*p* ≥ 0.05). Mean values in rows corresponding to the same sensory indicator marked with different capital superscript differ significantly (*p* < 0.05). ^∗^OSQ: overall sensory quality.

**Table 2:** Effect of drying and storage in MAP (20% CO_2_/80% N_2_) at 4°C and 20°C on CIE-LAB colour parameters of sausage snack.

Storage time (days)	Storage temperature							
	4°C				20°C			
	*L* ^∗^	*a* ^∗^	*b* ^∗^	Δ*E*	*L* ^∗^	*a* ^∗^	*b* ^∗^	Δ*E*
	Sausage							
	58.1 ± 0.53^a^	6.4 ± 0.33^a^	12.6 ± 0.12^ac^	19.35	58.1 ± 0.53^a^	6.4 ± 0.33^a^	12.6 ± 0.12^a^	19.35
	Sausage snack							
0	38.8 ± 0.42^b^	7.2 ± 0.15^b^	11.5 ± 0.38^b^	—	38.8 ± 0.42^b^	7.2 ± 0.15^b^	11.5 ± 0.38^b^	—
3	39.1 ± 0.65^b^	7.4 ± 0.12^b^	11.3 ± 0.39^b^	0.41	38.6 ± 0.56^b^	7.6 ± 0.15^c^	11.5 ± 0.80^b^	0.81
7	39.4 ± 0.90^bd^	6.9 ± 0.65^ab^	11.3 ± 0.49^b^	0.73	38.2 ± 0.74^b^	7.5 ± 0.27^d^	12.2 ± 0.90^b^	0.97
14	40.1 ± 1.06^b^	7.2 ± 0.68^b^	11.1 ± 0.32^b^	1.32	40.4 ± 0.89^c^	7.5 ± 0.45^d^	12.0 ± 0.46^b^	1.92
28	40.3 ± 0.99^b^	7.2 ± 0.53^b^	11.5 ± 0.81^b^	1.46	40.9 ± 0.39^c^	6.9 ± 0.27^e^	12.2 ± 0.72^b^	2.23
56	41.7 ± 0.95^c^	8.0 ± 0.52^bcA^	12.7 ± 0.51^acA^	3.23	43.7 ± 0.93^e^	5.9 ± 0.16^fB^	14.2 ± 0.55^cB^	5.76
70	43.8 ± 0.91^d^	7.8 ± 0.33^cA^	13.8 ± 0.29^dA^	5.51	45.4 ± 0.81^f^	5.9 ± 0.15^fB^	16.2 ± 0.47^dB^	8.12

Mean values in columns marked with the same superscript do not differ significantly (*p* ≥ 0.05). Mean values in rows corresponding to the same coordinate marked with different capital superscript differ significantly (*p* < 0.05).

**Table 3:** Effect of drying and storage in MAP (20% CO_2_/80% N_2_) at 4°C and 20°C on texture parameters of sausage snack.

Storage time (days)	Storage temperature							
	Hardness (*N*)	Springiness	Cohesiveness	Chewiness (*N*)	Hardness (*N*)	Springiness	Cohesiveness	Chewiness (*N*)
	Sausage							
	42.1 ± 2.8^a^	0.91 ± 0.05^a^	0.61 ± 0.06^a^	23.4 ± 2.9^a^	42.1 ± 2.8^a^	0.91 ± 0.05^a^	0.61 ± 0.06^a^	23.4 ± 2.9^a^
	Sausage snack							
0	162.7 ± 11.0^b^	0.79 ± 0.05^b^	0.65 ± 0.06^ab^	84.1 ± 12.9^b^	162.7 ± 11.0^b^	0.79 ± 0.05^b^	0.65 ± 0.03^a^	84.1 ± 12.9^b^
3	160.6 ± 11.5^b^	0.81 ± 0.04^b^	0.64 ± 0.06^ab^	82.2 ± 7.2^b^	165.8 ± 17.6^b^	0.75 ± 0.03^b^	0.66 ± 0.03^a^	82.3 ± 10.7^b^
7	163.5 ± 13.3^b^	0.82 ± 0.04^b^	0.68 ± 0.03^b^	91.4 ± 9.9^cA^	153.1 ± 17.2^b^	0.77 ± 0.04^b^	0.65 ± 0.03^a^	76.6 ± 12.8^bB^
14	175.5 ± 15.3^c^	0.81 ± 0.03^b^	0.65 ± 0.04^ab^	93.7 ± 11.5^c^	163.9 ± 13.5^b^	0.77 ± 0.06^b^	0.64 ± 0.04^a^	81.7 ± 11.3^b^
28	163.8 ± 13.5^b^	0.78 ± 0.04^c^	0.64 ± 0.03^ab^	81.7 ± 11.3^b^	160.8 ± 18.0^b^	0.78 ± 0.05^b^	0.60 ± 0.04^a^	77.2 ± 8.5^b^
56	181.3 ± 13.3^c^	0.81 ± 0.03^b^	0.65 ± 0.04^ab^	95.6 ± 8.9^c^	173.2 ± 17.6^b^	0.77 ± 0.06^b^	0.63 ± 0.04^a^	85.2 ± 13.1^b^
70	178.2 ± 19.2^c^	0.77 ± 0.05^c^	0.62 ± 0.04^aA^	84.9 ± 12.5^bA^	187.8 ± 25.0^c^	0.82 ± 0.06^c^	0.78 ± 0.05^bB^	100.7 ± 16.2^cB^

Mean values in columns marked with the same superscript do not differ significantly (*p* ≥ 0.05). Mean values in rows corresponding to the same texture parameter marked with different capital superscript differ significantly (*p* < 0.05).

**Table 4:** Effect of drying and storage in MAP (20% CO_2_/80% N_2_) at 4°C and 20°C on fat quality indicators of sausage snack.

Storage time (days)	Storage temperature					
	4°C			20°C		
	Acid value (mg KOH·g^−1^)	Peroxide value (meq O_2_·kg^−1^)	TBARS (mg MDA·kg^−1^)	Acid value (mg KOH·g^−1^)	Peroxide value (meq O_2_·kg^−1^)	TBARS (mg MDA·kg^−1^)
	Sausage					
	3.54 ± 0.06^a^	0.32 ± 0.01^a^	0.47 ± 0.05^a^	3.54 ± 0.06^a^	0.32 ± 0.01^a^	0.47 ± 0.05^a^
	Sausage snack					
0	3.05 ± 0.03^b^	0.54 ± 0.02^b^	0.51 ± 0.04^a^	3.05 ± 0.03^b^	0.54 ± 0.02^b^	0.51 ± 0.04^a^
3	3.05 ± 0.06^b^	0.63 ± 0.04^c^	0.75 ± 0.03^bA^	3.07 ± 0.04^b^	0.71 ± 0.05^c^	0.82 ± 0.04^bB^
7	3.09 ± 0.06^b^	0.64 ± 0.04^cA^	1.18 ± 0.03^cA^	3.10 ± 0.06^b^	0.75 ± 0.04^cB^	1.35 ± 0.03^cB^
14	3.07 ± 0.13^b^	0.65 ± 0.03^cA^	1.30 ± 0.04^dA^	3.12 ± 0.09^b^	0.84 ± 0.02^dB^	1.38 ± 0.04^cB^
28	3.18 ± 0.06^bA^	0.72 ± 0.03^dA^	1.43 ± 0.03^e^	3.48 ± 0.11^aB^	0.93 ± 0.02^eB^	1.47 ± 0.03^d^
56	3.46 ± 0.09^aA^	0.96 ± 0.03^eA^	1.49 ± 0.05^ef^	4.15 ± 0.11^cB^	1.37 ± 0.03^fB^	1.49 ± 0.05^e^
70	3.52 ± 0.06^aA^	1.17 ± 0.03^fA^	1.55 ± 0.07^fA^	4.19 ± 0.13^cB^	1.75 ± 0.03^gB^	1.66 ± 0.06^fB^

Mean values in columns marked with the same superscript do not differ significantly (*p* ≥ 0.05). Mean values in rows corresponding to the same indicator marked with different capital superscript differ significantly (*p* < 0.05).

**Table 5:** Effect of drying and storage in MAP (20% CO_2_/80% N_2_) at 4°C and 20°C on biogenic amine concentrations and BAI (mg kg^−1^) in sausage snack.

Storage temperature (°C)	Sausage	Sausage snack storage time (days)						
		0	3	7	14	28	56	70
	2-Phenylethylamine							
4	nd	nd	nd	nd	nd	nd	nd	nd
20			nd	nd	nd	nd	9.9 ± 0.27	10.2 ± 0.54
	Cadaverine							
4	nd	nd	nd	4.5 ± 0.23	4.5 ± 0.18	4.5 ± 0.29	4.4 ± 0.21	4.4 ± 0.18^A^
20			nd	4.5 ± 0.15^a^	4.5 ± 0.25^a^	4.4 ± 0.13^a^	4.5 ± 0.35^a^	5.0 ± 0.24^bB^
	Histamine							
4	10.7 ± 0.11^a^	18.4 ± 0.07^bc^	18.3 ± 0.05^c^	18.3 ± 0.04^c^	18.4 ± 0.05^c^	18.4 ± 0.02^bc^	18.4 ± 0.08^bc^	18.5 ± 0.12^bA^
20		18.4 ± 0.07^b^	18.4 ± 0.03^b^	18.4 ± 0.14^b^	18.5 ± 0.09^b^	18.5 ± 0.11^b^	18.6 ± 0.15^b^	19.1 ± 0.67^cB^
	Putrescine							
4	9.4 ± 0.41^a^	14.9 ± 0.67^b^	14.8 ± 0.49^b^	14.9 ± 0.91^b^	14.9 ± 0.86^b^	15.1 ± 0.71^b^	15.1 ± 0.89^b^	15.9 ± 0.71^bA^
20		14.9 ± 0.67^ab^	15.1 ± 0.46^ab^	15.4 ± 0.66^a^	15.1 ± 0.76^a^	15.4 ± 0.83^a^	16.0 ± 0.57^a^	17.2 ± 0.43^cB^
	Spermidine							
4	8.5 ± 0.5^a^	15.1 ± 0.2^b^	15.3 ± 0.2^bc^	15.1 ± 0.2^b^	15.0 ± 0.2^b^	14.9 ± 0.3^b^	15.3 ± 0.4^bc^	15.7 ± 0.0^cA^
20		15.1 ± 0.2^b^	15.9 ± 0.3^cd^	15.1 ± 0.6^b^	15.1 ± 0.2^bc^	15.5 ± 0.3^bc^	15.5 ± 0.5^bc^	16.2 ± 0.2^cdB^
	Spermine							
4	21.9 ± 0.72^a^	30.9 ± 0.60^b^	27.3 ± 0.62^c^	27.4 ± 0.57^c^	26.2 ± 0.66^d^	26.4 ± 0.20^d^	27.9 ± 0.85^c^	29.4 ± 0.55^e^
20		30.9 ± 0.60^b^	27.4 ± 0.54^c^	27.2 ± 0.91^c^	26.6 ± 0.87^c^	26.3 ± 0.81^c^	28.2 ± 0.74^c^	29.9 ± 0.85^b^
	Tryptamine							
4	6.8 ± 0.27^a^	10.1 ± 0.51^b^	9.3 ± 0.07^c^	9.3 ± 0.58^bc^	9.2 ± 0.50^bc^	9.4 ± 0.22^bc^	9.0 ± 0.08^dA^	8.8 ± 0.16^dA^
20		10.1 ± 0.51^b^	9.3 ± 0.13^b^	9.6 ± 0.70^b^	9.5 ± 0.23^b^	9.5 ± 0.20^b^	9.6 ± 0.07^bB^	9.6 ± 0.16^bB^
	Tyramine							
4	17.4 ± 0.07^a^	24.8 ± 0.12^b^	24.9 ± 0.16^b^	29.2 ± 0.57^c^	28.8 ± 0.82^c^	28.7 ± 0.50^cA^	29.1 ± 0.30^bA^	28.5 ± 0.55^bA^
20		24.8 ± 0.12^b^	25.1 ± 0.07^b^	29.5 ± 0.56^c^	28.7 ± 0.26^c^	31.2 ± 0.70^dB^	31.9 ± 0.30^dB^	32.1 ± 0.48^dB^
	BAI							
4	34.5	58.1	58.0	66.9	66.6	66.7	67.0	67.3
20			58.6	67.8	66.8	69.5	71.0	73.4

Mean values for particular amine marked with the same superscript or not marked at all in the row do not differ significantly (*p* ≥ 0.05). Mean values for particular amine in the column marked with different capital letters differ significantly (*p* < 0.05).

## Data Availability

The data used to support the findings of this study are available from the corresponding author upon request.
